# The importance of human population characteristics in modeling *Aedes aegypti* distributions and assessing risk of mosquito-borne infectious diseases

**DOI:** 10.1186/s41182-017-0078-1

**Published:** 2017-11-15

**Authors:** Julie F. Obenauer, T. Andrew Joyner, Joseph B. Harris

**Affiliations:** 1 Department of Epidemiology and Biostatistics, College of Public Health, East Tennessee State University, Johnson City, TN USA; 2 Department of Geosciences, Geoinformatics and Disaster Science Lab, College of Arts and Sciences, East Tennessee State University, Johnson City, TN USA; 3 Department of Geography & Anthropology, College of Humanities and Social Sciences, Louisiana State University, 227 Howe-Russell-Kniffen Geoscience Complex, Baton Rouge, LA 70803 USA

**Keywords:** *Aedes aegypti*, Zika virus, Climate change, Species distribution model, Maxent, Human population, Mosquito distributions

## Abstract

**Background:**

The mosquito *Aedes aegypti* has long been a vector for human illness in the Southeastern United States. In the past, it has been responsible for outbreaks of dengue, chikungunya, and yellow fever and, very recently, the Zika virus that has been introduced to the region. Multiple studies have modeled the geographic distribution of *Ae*. *aegypti* as a function of climate factors; however, this ignores the importance of humans to the anthropophilic biter. Furthermore, *Ae*. *aegypti* thrives in areas where humans have created standing water sites, such as water storage containers and trash. As models are developed to examine the potential impact of climate change, it becomes increasingly important to include the most comprehensive set of predictors possible.

**Results:**

This study uses Maxent, a species distribution model, to evaluate the effects of adding poverty and population density to climate-only models. Performance was evaluated through model fit statistics, such as AUC, omission, and commission, as well as individual variable contributions and response curves. Models which included both population density and poverty exhibited better predictive power and produced more precise distribution maps. Furthermore, the two human population characteristics accounted for much of the model contribution—more so than climate variables.

**Conclusions:**

Modeling mosquito distributions without accounting for their dependence on local human populations may miss factors that are very important to niche realization and subsequent risk of infection for humans. Further research is needed to determine if additional human characteristics should be evaluated for model inclusion.

## Background

As the 1963 training manual on “Mosquitoes of Public Health Importance and Their Control” puts it, “Mosquitoes have probably had a greater influence on human health and well-being throughout the world than any other insects” [1]. This is particularly true in the USA for the mosquito *Aedes aegypti*, the vector for Zika virus, dengue, chikungunya, and yellow fever. It has long been, and continues to be, the focus of much of the local concern for human health [2–4]. Furthermore, the Southeastern region has historically borne the brunt of these infectious diseases when they appear within the conterminous USA. The only outbreak of chikungunya in the USA occurred in Florida in 2014 and, before that, dengue was spread locally in Texas in 2005 [5, 6]. Historically, yellow fever caused several major outbreaks in the Southeast, particularly along the Mississippi River [7], with the last major outbreak occurring in New Orleans in 1905 [8]. And, now the USA is bracing itself for the spread of Zika virus from Central and South America into the Southeast [9].

Mosquitoes have specific habitat requirements that vary based on each particular species. For *Ae*. *aegypti*, the preferred habitat is in tropical and subtropical climates, but a recent study estimated that the current distribution does extend into some temperate regions [10]. While *Ae*. *aegypti* is sensitive to temperature, other climatic factors such as precipitation and altitude-influenced proxies are important as well [4, 11]. However, a close examination of the life cycle of this mosquito species also reveals that anthropogenic factors may strongly influence their geographic distribution. *Ae*. *aegypti* is a species that rests indoors and takes its blood meals primarily from humans [12]. It also lays its eggs in containers of standing water, which are often found near the humans who use those containers to store their water [13]. Poverty may be an indicator of areas with increased amounts of standing water, due to a lack of local sanitary services. Poverty may also portend a lack of air conditioning, which would raise the need for opening windows, thereby increasing mosquito entry. As these examples show, many of the predictors of mosquito-human interactions are affected by socioeconomic status within an area [4, 14, 15].

Previous modeling efforts focused primarily on temperature and other climatic variables for niche identification in *Ae*. *aegypti* distribution models. Campbell et al. [16] modeled *Ae*. *aegypti* as a vector for chikungunya and dengue globally and their models restricted parameters entirely to climatic variables, and Brady et al. [17] only examined temperature as a predictor of vector competence for dengue. In modeling the distribution of *Ae*. *aegypti* globally using boosted tree regression models, Kraemer et al. [4] explored the contributions of enhanced vegetation indices, climatic variables, and urbanization. To model urbanization, they generated a categorical variable from the Global Rural Urban Mapping Project (GRUMP), population density metrics, and night-time satellite imagery displaying land usage. The categories created consisted of urban, peri-urban, and rural classifications. They found a relatively small effect of urban designation on habitat suitability but hypothesized that satellite data showing human habitats may be more informative [4]. However, each of these modeling attempts identified roughly the same geographic regions within the Southeastern United States as suitable for *Ae*. *aegypti*, but while it is informative to know where the mosquito can live, this does not help identify where individuals are at the highest risk of interactions with mosquitoes.

To understand the full picture of niche suitability and appropriately model the risk of mosquito-human interaction, it is necessary to model possible niches as a function of both bioclimatic and human population characteristics. As Eisen and Moore [14] indicated, understanding how temperature and precipitation affect suitability does not account for factors that may confound the relationship between niche suitability and niche realization. Restricting models to climate variables, while convenient, oversimplifies the actual niche dynamics of the species [15]. This problem is magnified when models are projected for climate change. Human population is changing, both due to population expansion and the impacts of climate change and conflict-related migration [18, 19]. It is unreasonable to assume this will not affect the interactions between humans and mosquitoes.

To address this divide, this analysis seeks to examine how human population characteristics impact species distribution modeling for *Ae*. *aegypti*. This analysis will use bioclimatic variables identified in previous modeling efforts [16] for an initial model. Then, additional variables that account for population density, as a measure of availability of feeding opportunities, and poverty, as a surrogate for likelihood of human-mosquito interaction, will be added and tested for model performance and potential improvement.

While *Ae*. *aegypti* is present in a variety of global locations, modeling a small and well-characterized region can increase the predictive capabilities of a model [15], which should be more appropriate here, in testing variable contribution to model fit. Since the Southeastern United States is highly vulnerable to *Ae*. *aegypti* and has well-catalogued climate, population density, and poverty data, this region will be used to test the relative effects of these variables on the model. The results of this model will inform future *Ae*. *aegypti* models with respect to the importance of human population characteristics.

## Methods

### Data

Occurrence data for *Ae*. *aegypti* was taken from the dataset available through the “Global Compendium of *Aedes aegypti* and *Aedes albopictus* Occurrence” [10]. This compendium was based primarily on a review of published occurrence datasets, but also included surveillance system data, national mosquito surveys, and records from governmental health agencies, where available. All occurrences were recorded between 1960 and 2014 and contained latitude and longitude for georeferencing [10]. Since the dataset contains occurrences with georeferencing and metadata information but no information on sampling or absence, this is a presence-only dataset. To avoid issues common to “opportunistic” sampling (i.e., oversampling of more accessible and higher risk areas), Kraemer et al. [10] applied methods of spatial and temporal standardization. For example, a single occurrence was defined as an observation within one calendar year at a given unique location (5 km^2^). This resulted in 1112 occurrences of *Ae*. *aegypti* being removed from the global compendium dataset [10].

Publicly available data from WorldClim.org were used for current bioclimatic variables. This dataset contains 19 variables known as the “bioclim” set, which includes characteristics designed to model annual trends, such as annual mean temperature, isothermality, mean temperature in warmest and coldest quarters, and mean precipitation in wettest and driest quarters. The bioclim variables are calculated from other variables within the set, meaning that multicollinearity is a concern [20]. To determine which variables should be included, previous modeling efforts were consulted. Bio8 (mean temperature of wettest quarter), bio9 (mean temperature of driest quarter), bio18 (precipitation of warmest quarter), and bio19 (precipitation of coldest quarter) were omitted from the analysis due to artifacts in the data found in similar research [16]. The WorldClim climate surfaces were calculated based on average observations from field sites taken between 1960 and 1990. Data were interpolated across regions with few observation sites. However, data coverage in the USA, and particularly the Southeastern portion, were extensive and needed only minimal interpolation, therefore reducing associated surface error [20].

Population density was accessed through the Socioeconomic Data and Applications Center (SEDAC), which makes global socioeconomic data publicly available in formats that can be used in ArcGIS. In this case, the Population Density Grid v.3 data were downloaded in ascii format. Data represented the population density of the USA in the year 2000 to match occurrence and climate data and were displayed as population count/land area with a unit of persons per square kilometer. The data were calculated and displayed at 2.5 arc minutes [21]. Because *Ae*. *aegypti* is highly anthropophilic, population density data are very important to include in model development and comparisons.

To model poverty, a factor that strongly influences an area’s environmental conditions, a raster surface from the 2000 US Census was added. Data were displayed at 30 arc seconds (~ 1 km^2^) but resampled at the resolution of the population density grids. The poverty variable was “Proportion of population living below the poverty level” for the continental USA in 2000 [22]. Poverty levels for individuals were defined as annual incomes at or below $8350 and $17,050 for a family of four. Also, taken from the US Census was a regional shapefile for the Southeast to use as the extent and a state boundaries shapefile for visual reference [23, 24].

### Maxent

The Maxent model was chosen for this analysis because it is designed for presence-only data and known to outperform other common presence-only models [25]. The method uses presence-only data to identify cells in which the species has been found. It then samples the user-provided predictors, in this case climate and human population characteristics, to identify other areas that match the presence areas of these predictors. Since occurrences that are geographically close are likely to exhibit spatial autocorrelation, Maxent requires rarefication (reducing the number of occurrences to no more than one per cell) prior to model implementation and the resulting output is a relative probability surface. It is important to remember that Maxent output should be interpreted as the probability of a location containing a suitable niche, not the probability of species presence [26, 27]. Finally, while global modeling efforts are common, Maxent may perform better when smaller geographical regions are modeled [25], which is particularly relevant in this paper.

### Processing

**Fig. 1:**
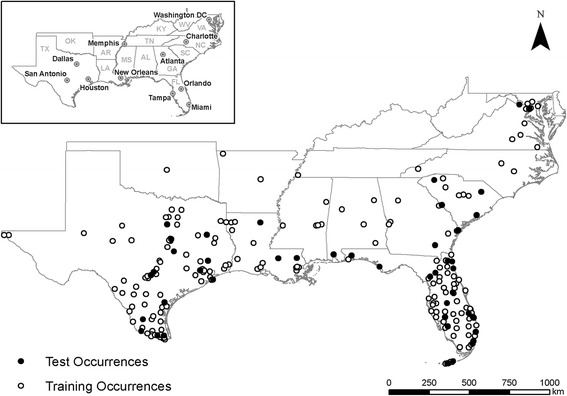
Occurrence data for *Ae*. *aegypti* split into training and testing subsets

All data were processed in ArcMap 10.3. Initially, the population density raster was cropped using the Southeast region shapefile as a mask. Then, climate and poverty rasters were resampled at the resolution and extent of the population density raster, which meant all data were converted to the resolution of 2.5 arc minutes (~ 5 km^2^). Species occurrence data were rarefied in ArcMap then split into testing and training sets (Fig. 1). The split set contained 80% of the points for training (181) and 20% for testing (45), which is based on a heuristic formula that suggests 1/(1−√(*p−*1)) where *p* is the number of predictors used in the model [28]. Post processing was also done in ArcMap.

The Maxent software version 3.3.3k was used for all modeling efforts. This is a GUI that requires the user to select the species training file and the environmental layers. Users are given several options for model output, and we chose to create response curves and perform jackknife analysis to test variable importance. Additionally, the file for the 20% testing dataset was specified and the model threshold was set at 10 percentile training presence, meaning that models which excluded more than 10% of training observations were rejected [29]. Additional model settings comprised the inclusion of occurrence data within the background sampling process. This process replaces the uniform background data that is randomly sampled and instead creates a background dataset drawn from the distribution of the occurrence data [29]. Maximum entropy distribution is then selected relative to the provided background, effectively eliminating selection bias. In addition to response curves, variable jackknifing, and variable gain values, Maxent outputs a probability surface ranging from 0.0 to as high as 1.0.

The Maxent model was run in four iterations. The first was climate only, as a reference for models with additional parameters. Then, models were run with climate and poverty, and again with climate and population density to assess how the addition of each predictor individually affected the model. The final model included the original climate variables and both poverty and population density. Model evaluation depended on the AUC, omission error, and commission. Omission error was calculated based on omission of test data (20% excluded from the model) at the 10-percentile training presence level, so once a model was produced based on the inclusion of at least 90% of training data, the test data were overlaid and all data that were found in areas of 0.5 logistic probability or less (< 50% likelihood of suitability) were deemed omitted. Commission was calculated as the percent of total study area predicted to have a probability greater than 0.5, also at the 10-percentile training presence level. The selection of a threshold is a topic of much debate (e.g., [30, 31]), and for our study, we selected a threshold (0.5) that maximizes the percent of points correctly classified (> 90%). This is a modification of the lowest presence threshold method [32]. Models were ranked by order of AUC, so long as AUC was higher than 0.6 (0.5 means a model’s predictions are effectively random) [28]. Individual components were also evaluated under jackknife and response curve results to determine how much each predictor contributed to the model. Additionally, AUC scores were compared between models to test for significant increases in improvement. To test for statistical significance, standard errors were obtained for each model (based on number of samples and model standard deviation), then a two-sample *z*-statistic was calculated using the difference of AUC values and standard errors between each model pairing.

## Results

The inclusion of human population characteristics improved model fit (see Table 1). Each model produced a maximum entropy logistic output, following the formula *c* × *r*/(1 + *c* × *r*) where *c* is the exponential of the entropy of maximum distribution and *r* is the raw value corresponding to a logistic value. The training AUCs for the models testing climate and poverty (model 2) and climate and population density (model 3) were comparable with each other but higher than those for the climate-only model. The full model, which included climate, population density, and poverty, had slightly higher training AUCs (0.922) than model 2 (0.914) or model 3 (0.919). The omission error was reduced by the addition of the population characteristics. The omission for model 3 and the full model was the same at 4.5%, but this was less than half of the climate-only omission rate of 9.1%. The addition of population density in model 3 resulted in the most significant reduction in commission, going from 33.4% in the climate-only model to 20.6%. Adding poverty in the full model increased the commission very slightly above that of model 3.

**Table 1:** Model fit statistics for all four models

	Climate-only model	Model 2: climate + poverty	Model 3: climate + population density	Full model: climate + population density + poverty
Training AUC	0.880	0.914	0.919	0.922
Omission	9.1%	6.8%	4.5%	4.5%
Commission	33.4%	26.7%	20.6%	21.0%

**Fig. 2:**
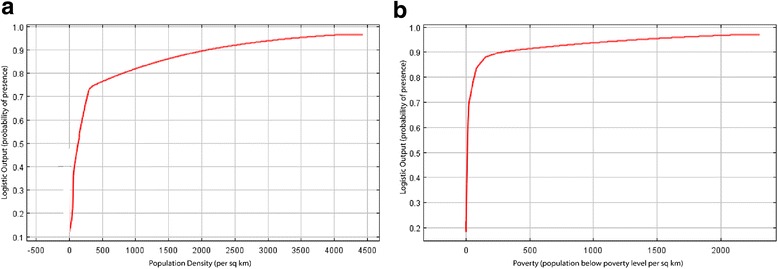
Response curves for (**a**) population density (glds00ag) and (**b**) poverty (uspov00) in the full model. The response curve for population density indicates a rapid increase in suitable environments from 0 to 300 people per square kilometer, with a gradual increase occurring in areas with population densities greater than ~300 people per square kilometer.  A similar trend is exhibited by the poverty response curve, although the response curve begins to plateau around 100 people per square kilometer living below the poverty level

The response curves for the full model (Fig. 2) show that there is an increasing probability of presence as both poverty and population density increase. However, for poverty, the increase is rapid at first, then relatively flat, with very low levels of poverty increasing the probability to 90% and higher levels resulting in a gradual increase to above 95%. Population density exhibited a steadier rise, with an increase from 75% probability of prevalence at 50 people per km^2^ to 95% probability at 4500 people per km^2^. For the response curves, it should be noted that a value of less than zero on the *X* axis is not meaningful for these variables.

Population density was also the single largest contributor in the full model at 43.7%. Poverty was fourth at 11%, behind bio1 (mean annual temperature) at 15.4%, and bio6 (minimum temperature of the coldest month) at 14.6%. The jackknife analysis for the training set of occurrences showed that population density contributed the most useful information to the model, followed by the poverty variable.

**Fig. 3:**
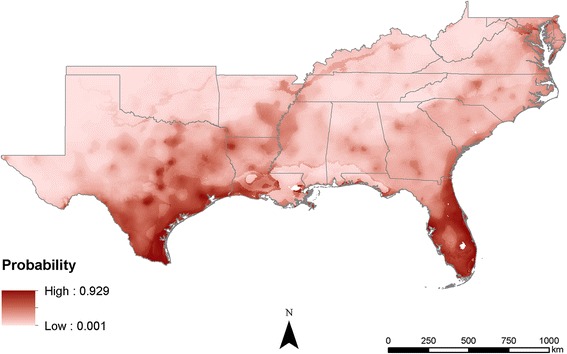
Maxent probability surface output for model 1—climate-only model

**Fig. 4:**
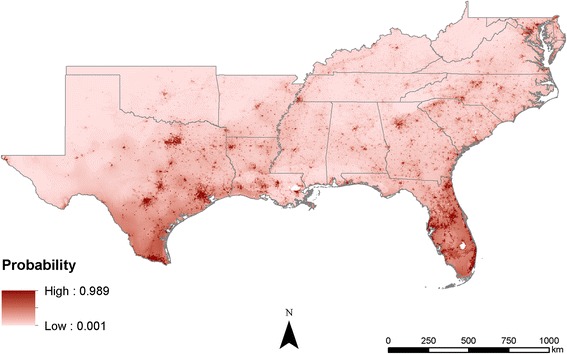
Maxent probability surface output for model 2—climate + poverty

**Fig. 5:**
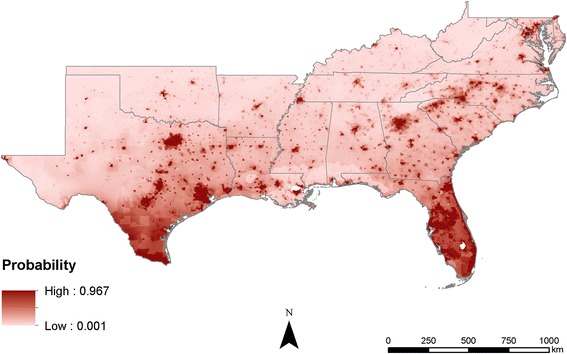
Maxent probability surface output for model 3—climate + population density

**Fig. 6:**
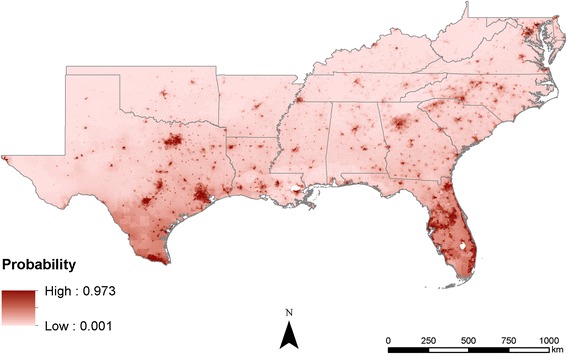
Maxent probability surface output for full model

Comparing the output maps shows that the probabilities estimated become increasingly precise as more information is added. In the climate-only model, the estimates are broad swaths of cells that smoothly transition from one probability to another (Fig. 3). As the human population characteristics are added, less smoothing occurs across the predicted surfaces, likely due to the added information about humans (Figs. 4 and 5). The interactions of poverty, population density, and climate lead the final model to highlight major population centers (Fig. 1) such as Atlanta, as well as areas that are highly populated and within the known range of *Ae*. *aegypti*, like much of Florida (Fig. 6).

Further, each model pairing was tested for significant improvement based on the AUC scores (Table 2). Common methods for comparing AUC scores are described by DeLong et al. [33] and Hanley and McNeil [34]. The mean AUC difference between two models was divided by the square root of the squared sum of each model’s standard error. Results showed that the largest statistical improvement (larger *z*-scores) occurred between the climate-only model and the full model, followed by the climate-only model and the climate + population density model and the climate-only model and the climate + poverty model. While the statistical improvement was much smaller between the other model pairings, the differences remained significant (*p* < 0.01).

**Table 2:** Tests for significant improvement between each model

Model pairing	Mean AUC differences	*z*-scores
Climate only and climate + poverty	0.034	0.203^*^
Climate only and climate + pop. density	0.039	0.244^*^
Climate only and full model	0.042	0.263^*^
Climate + poverty and climate + pop. density	0.005	0.038^*^
Climate + poverty and full model	0.008	0.060^*^
Climate + pop. density and full model	0.003	0.024^*^

*p < 0.01

## Discussion

The model fit indices for these models clearly demonstrate that the addition of poverty and population density improves the model’s predictive power. Additionally, while each predictor performs well when added independently, the best fitting and most accurate model is the full model, with both human-interaction predictors included. Investigating variable contributions and response curves supports this finding. The variable that contributes the most information varies depending on which model fit index is being discussed, meaning that both make valuable contributions to the overall model. While human-interaction predictors proved to be highly influential for the models and AUC scores showed significant differences, AUC scores alone were fairly high for all models. This indicates that AUC scores alone provide an incomplete picture, as the modeled surfaces appeared to be quite different between the climate-only model and the human-interaction models.

These findings support the assertion that models which only explore climate variables, such as temperature and precipitation, are incomplete and may be missing a significant source of information on habitat suitability. This is particularly highlighted in Maxent when considering that population density contributed almost three times more information than the next predictor, mean annual temperature. Since *Ae*. *aegypti* is highly anthropophilic in its feeding preferences and relies on standing water for breeding, it is logical that human population characteristics have proven to be important in modeling possible habitats and high-risk areas. While much of the Southeast United States may present a suitable habitat for *Ae*. *aegypti*, this study identified *preferred* habitat for the mosquito, and thus areas where *Ae*. *aegypti* is more likely to spread various diseases, including Zika. Variables used for this study, especially poverty and population density, may infer an observation/sampling bias for mosquitoes, but the mosquito locality database [10] applied both spatial and temporal methods of standardization to reduce sample bias, and our own process of rarefication further reduced any sampling bias that may be more related to abundance (i.e., number of observations in densely populated areas). Additionally, the union of occurrence data with background samples eliminated bias associated with random background sampling, resulting in a Gibbs distribution that accounted for bias in the final predictions [29].

Interestingly, the addition of human population characteristics illustrates that probability surfaces become more a probability of human-mosquito interaction that leads to niche realization than a probability of suitability or presence. This is an important distinction in understanding the risk mosquitoes pose to human health, particularly when seeking to implement mosquito control programs in regions that are at the highest risk of mosquito-borne illnesses. A high probability of suitability in these maps still does not guarantee species presence, but it does show that there is a high likelihood that the combination of ideal climatic conditions and human population characteristics provides suitable conditions for feeding and breeding. In many of the northern states within the study region (e.g., Kentucky, Tennessee, Oklahoma), occurrences of *Ae*. *aegypti* have thus far been minimal, but models indicate that many elements of a suitable habitat, and consequently a higher risk of mosquito-borne illnesses, are present, especially in the larger urban environments. Long-term changes in climate in these regions may result in an increased risk to mosquito-borne illnesses as mosquitoes establish populations at higher latitudes [4].

This research also indicates that, as Eisen and Moore [14] feared, there is the possibility of human population characteristics confounding the relationship between climate and mosquito presence. This is important considering the on-going focus on modeling both current species presence and changes in habitats due to climate change. As climate change alters the environmental landscape, it will also affect the human landscape [19]. Additionally, human population dynamics will change on their own, further altering the manmade landscape, in terms of population characteristics and built environment [18]. Failing to account for these changes may likely result in only a partial understanding of future species expansion, especially since so much of the current model’s explanatory power is due to population density.

Exploring human characteristics in climate projections currently faces some problems. For example, WorldClim projects climate change to the year 2050 but SEDAC only projects population density to 2030. Another issue is that poverty data were obtained from the US Census and not every country has data that is so complete or reliable. Therefore, adding human population characteristics to global maps or projections is problematic and requires data that may be difficult to obtain. Additional environmental parameters may be useful and could be compared to models that used population density (e.g., an enhanced vegetation index (EVI)). The EVI, or similar satellite-derived variables, may serve as acceptable proxies to population density.

An important limitation to consider in assessing these models is the lack of literature on which human population characteristics are most important. Poverty was included as a surrogate for likelihood of being in an environment that is suitable for mosquitoes (i.e., has untreated standing water) and population density was included since *Ae*. *aegypti* is highly anthropophilic. However, there may be more suitable surrogates for these characteristics or, for areas where it is available, measuring the presence of standing water may be a much better predictor. It is clear from these results that significantly more research is needed to better understand which human characteristics increase niche suitability (or preference) for vector mosquitoes and how to model those interactions.

## Conclusions

Mosquito distributions have been modeled and predicted globally, regionally, and locally using many different methods and datasets; however, applying the concept of “human risk” to infection has not been well studied. In many cases, mosquito populations depend on local human populations and our inclusion of human population density in distribution models resulted in risk probability surfaces, not species distribution probabilities. This is an important distinction in understanding the models and is crucial for niche realization and subsequent risk of infection for humans. While the models (Figs. 4, 5, and 6) not surprisingly follow population density patterns, many cities, especially those in the northern extent of the study area (e.g., Nashville and Oklahoma City), show a higher risk probability when compared to the climate-only model (Fig. 3). Further research is needed to determine if additional human characteristics should be evaluated for model inclusion.
